# Climate change and the northern elephant seal (*Mirounga angustirostris*) population in Baja California, Mexico

**DOI:** 10.1371/journal.pone.0193211

**Published:** 2018-02-15

**Authors:** María C. García-Aguilar, Cuauhtémoc Turrent, Fernando R. Elorriaga-Verplancken, Alejandro Arias-Del-Razo, Yolanda Schramm

**Affiliations:** 1 Departamento de Oceanología Biológica, Centro de Investigación Científica y de Educación Superior de Ensenada, Ensenada, Baja California, México; 2 Departamento de Oceanología Física, Centro de Investigación Científica y de Educación Superior de Ensenad, Ensenada, Baja California, México; 3 Departamento de Pesquerías y Biología Marina, Centro Interdisciplinario de Ciencias Marinas del Instituto Politécnico Nacional, La Paz, Baja California Sur, México; 4 Departamento de Ciencias Químico Biológicas, Universidad de las Américas Puebla, Puebla, México; 5 Facultad de Ciencias Marinas, Universidad Autónoma de Baja California, Ensenada, Baja California, México; University of Arkansas, UNITED STATES

## Abstract

The Earth′s climate is warming, especially in the mid- and high latitudes of the Northern Hemisphere. The northern elephant seal (*Mirounga angustirostris*) breeds and haul-outs on islands and the mainland of Baja California, Mexico, and California, U.S.A. At the beginning of the 21st century, numbers of elephant seals in California are increasing, but the status of Baja California populations is unknown, and some data suggest they may be decreasing. We hypothesize that the elephant seal population of Baja California is experiencing a decline because the animals are not migrating as far south due to warming sea and air temperatures. Here we assessed population trends of the Baja California population, and climate change in the region. The numbers of northern elephant seals in Baja California colonies have been decreasing since the 1990s, and both the surface waters off Baja California and the local air temperatures have warmed during the last three decades. We propose that declining population sizes may be attributable to decreased migration towards the southern portions of the range in response to the observed temperature increases. Further research is needed to confirm our hypothesis; however, if true, it would imply that elephant seal colonies of Baja California and California are not demographically isolated which would pose challenges to environmental and management policies between Mexico and the United States.

## Introduction

The Earth′s climate is changing and increases in global surface temperatures attributable to human activities have become apparent since the 1950s, especially in the mid- and high latitudes of the Northern Hemisphere [1]. Pinnipeds are top predators that use extensive marine areas during their foraging trips, and climate change has been implicated in influencing their foraging success and at sea distribution [2–4]. However, because pinnipeds always return to pack ice or land to breed and haul-out, environmental changes to their terrestrial habitat also affects their distribution and abundance.

The effects of the decline in sea-ice on the abundance and demographic parameters of ice-associated pinnipeds had been well documented for some species, such as the Pacific walrus (*Odobenus rosmarus*) [5], and the ringed seal (*Phoca hispida*) [6], but climate change impacts on land-breeding pinnipeds is less well known. It has been suggested that a reduction in sea-ice could result in an increase in available coastal habitats of these species, allowing them to extend their distribution ranges toward the poles [7]. Alternately, the expected warmer climates could limit both range expansion and population growth because elevated air temperatures produce thermal stress and increase pup mortality [7].

**Fig 1:**
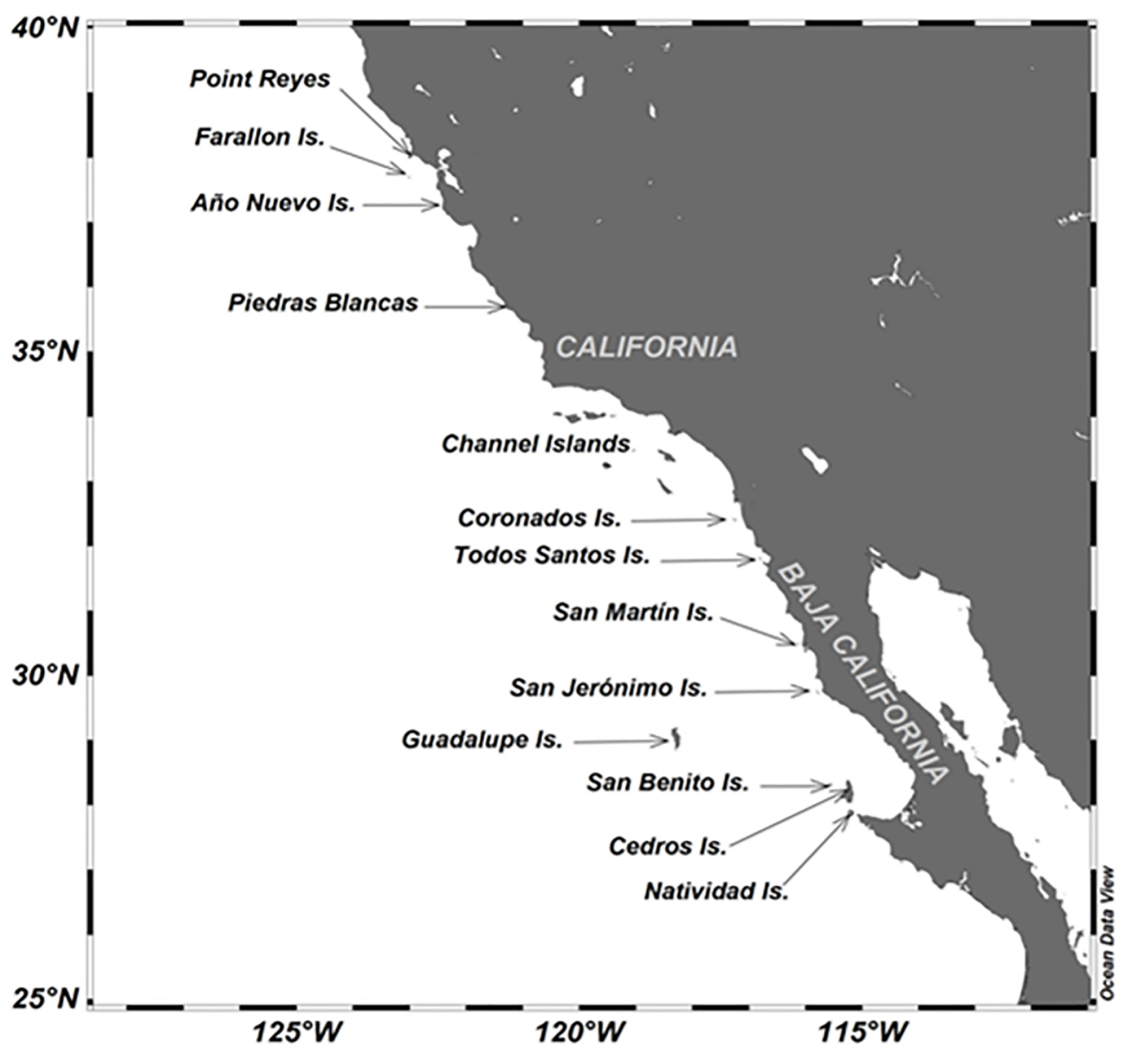
Breeding and haul-out sites of the northern elephant seal in California, USA, and Baja California, Mexico.

Northern elephant seals (*Mirounga angustirostris*) forage offshore of the North Pacific, in the Gulf of Alaska, and near the Aleutian Islands [8, 9]. Their annual cycle includes four terrestrial phases: the breeding season occurs in winter, weaned pups remain on land for more than two months (i.e. the post-weaning fast); in spring adult females and juveniles molt, in summer adult males molt; and in autumn juveniles haul-out [10]. The breeding and haul-out sites are located on islands and at some mainland locations in Baja California and California, U.S.A., in the subtropical eastern Pacific [10] (Fig 1) where mild temperatures are dominant throughout the year, winters are wet, and summers are warm and dry [11]. The climate in this region is influenced by both the California Current, which transports cold waters southward, and the California Counter-Current, which carries warm subsurface waters northward [12]. The region is highly susceptible to the oceanographic and climatic perturbations produced by El Niño/La Niña events, and a trend toward warmer winter and spring temperatures has been reported since the late 1950s [13].

The northern elephant seal was nearly eliminated by overhunting in the 19th century, and consequently suffered a severe population bottleneck that reduced its genetic diversity [14, 15, 16], likely making it highly vulnerable to environmental changes [17]. At the beginning of the 21st century, the California population was estimated around 179,000 individuals, and the average annual growth rate was estimated at 3.8% between 1988 and 2010 [18]. The current status of the Baja California population is unknown.

Main breeding sites of the northern elephant seal in Baja California are located in three insular areas: Guadalupe Island, San Benito Islands, and Cedros Island (Fig 1), which together represent >99% of the Baja California population [19]. Coronados and Todos Santos Islands (Fig 1) have been permanent breeding areas since the 1970s and 2000s, respectively, but the number of births is almost negligible, < 30 births/yr each [20, 21]. Some pups have occasionally been born on other coastal islands, such as Natividad, San Martín, and San Jerónimo (Fig 1) [21, 22], but no breeding groups have settled there.

Up until 1991, Baja California colonies appeared to be stable for a period of ~20 years, and at that time they were contributing ca. 25% of births of the whole northern elephant seal population [23]. However, in the past few decades, Guadalupe Island fishermen have noted a reduction in the number of seals that occupy breeding beaches of the island (F. Arellano pers. comm.), and a decrease in the San Benito colony has recently been reported [24].

We hypothesize that the elephant seal population of Baja California is experiencing a decline because the animals are not migrating as far south due to warming sea and air temperatures. In this paper, we assessed the status of Baja California colonies using counts of either live pups or adult females since 1965 at the three major sites, i.e., Guadalupe, San Benito, and Cedros, and analysed the climatic trends using sea surface temperature (SST) data spanning the last 120 years for the waters that surrounding the breeding range of the northern elephant seal. The SST was used as a climate indicator because of its direct influence on the lower atmosphere, and hence on local weather and climate [25]. Additional air temperature data recorded at Cedros Island since the late 1950s was also used to assess local climate trends.

## Materials and methods

### Status of the Baja California population

We used the number of pups produced to estimate both population trends and size since this is the best way to reduce bias in the assessment [26]. We compiled direct counts of live pups (suckling and weaners) conducted at the end of the birthing season in February, where we assumed the number of pups counted was equal to the number of pups produced (Table 1). Since pups remain on land until ~2.5 months old, we assumed that all living pups were detected. However, because counts were carried out at the end of the birthing season, it is likely that some pups had died before the count, and therefore the total pup production could be slightly underestimated. For the Baja California population, the mortality rate between birth and weaning has been estimated for a single colony (San Benito), and only for two seasons in the early 2000s [27]. We decided not to use that information to try to estimate pup production because this rate can vary annually and locally.

**Table 1:** 

Year	Guadalupe Island	San Benito Islands	Cedros Island	References
1965	3,668^a^	800^a^	0^a^	[28]
1969	7,104^a^			[23]
1970	5,520^a^	1,578^a^	49^a^	[23]
1975	6,058^a^	815^a^		[23]
1977	5,642^a^	1,359^a^	112^a^	[23]
1978	5,552^a^	1,727^a^		[23]
1980	5,011^a^	1,752^a^		[23]
1982	4,760^a^			[23]
1985			166^a^	[23]
1988			227^a^	[23]
1991	4,962^a^	1,662 ^a^	391^a^	[23]
2001		1,849 ^a^		[29]
2002		2,024 ^a^		[30]
2003		2,050 ^a^		[30]
2004		1,771 ^a^^,^^1^		
2005	3,785^a^^,^^1^		304^a^	[31]
2009	3,074^b^	1,689^b^	411^a^	[21]
2010	3,150^b^^,^^1^		339^a^^,^^1^	
2013		1,504^a^		[24]
2014		1,097^a^		[24]
2015	2,037^b^^,^^1^	1,205^a^		[24]
2016		1,317^a^^,^^1^		

Number of northern elephant seal pups produced in the Baja California colonies, 1965–2016.

^a^Direct counts of live pups (suckling and weaners) conducted in February.

^b^Estimated based on adult females counts conducted in January.

^1^Present study.

In some years, no pup counts were carried out on Guadalupe and San Benito islands, but direct counts of females were conducted in January. The number of pups produced was estimated based on these counts (Table 1) taking into account the temporal distribution of females on land together with the birth rate (*B*). Female elephant seals are asynchronous [32], but their temporal distribution on land remains consistent from year to year [27, 33]. Female temporal distributions were described for the San Benito colony [27] using the Rothery and McCann model (RMM) [33]. Thus, we calculated the total number of females that arrived at each colony during the whole breeding season as the number of females counted divided by the expected proportion of females on land at the count date, obtained based on the RMM. The number of pups produced was estimated by multiplying the total number of females by *B*, estimated at 74.19 ± 3.45% for the San Benito colony during the period 2002–2016 [34]. It is important to note that both the expected proportion of females on land and *B* were calculated for the San Benito colony, but were used to estimate the pup production in Guadalupe (see Table 1). This could represent a source of error since these values could vary locally; however, this was the only way to estimate the pup production with the available data.

The intrinsic rate of population increase was calculated by linear regression: log_e_ number of pups produced regressed on time, where *r* is the slope of the linear regression, and converted to the annual rate of increase as *λ* = *e*^*r*^ [35]. Because trends can vary in time, the optimal breakpoint in a time series with *n* ≥10 was determined using the freely available software SegReg (https://www.waterlog.info). The coefficient of variation (CV) of the average annual growth rate was calculated dividing the slope of the regression by the standard error of the slope [18].

Population size was estimated for 2009 because it was the latest year with available data from all colonies. The estimate was made by multiplying the number of pups produced by the factor *M* calculated for the northern elephant seal population in California [18], which varies according to the average annual rate of increase *λ*.

### Climate trends

We used monthly SST data for the Pacific Ocean basin, spanning the period January 1897 through December 2016, from the Extended Reconstruction Sea Surface Temperature Version 3b (ERSSTv3b). This version of the database has data available through 2016 [36], and is freely available from https://www.ncdc.noaa.gov. Prior to the estimation of SST trends, a low-pass Lanczos filter [37] with a cutoff period of 1.2 yr was applied to the data to remove the semi-annual and annual cycles. Rotated empirical orthogonal functions (REOF) were then calculated for the entire Pacific Ocean basin from the filtered and detrended data to isolate the El Niño Southern Oscillation (ENSO) signal. Rotation of the empirical orthogonal functions was done following the varimax method [38].

SST trends were estimated over 30 year periods beginning in 1897 from the residual of the filtered (but not detrended) data and a reconstruction of Pacific Ocean SSTs done using only the first REOF mode, which clearly captures the ENSO signal. The SST trends, estimated following Sen’s slope method [39], are therefore assured to reflect only variability on decadal—or longer—time scales. Statistical significance of the trends at the 95 and 99% confidence levels was evaluated by use of the nonparametric Mann-Kendall test (MK) [40].

Records of air temperature taken at Cedros Island during the period 1957–1995 were obtained from the Servicio Meteorológico Nacional (SMN) dataset, freely available at the Comisión Nacional del Agua (CONAGUA) web page. Data were recorded at SMN station 2027 (28°5’50″N, 115°11’12″W, elevation 10m). The datasets consist of daily maximum, minimum, and mean air temperature values. These data were used to estimate monthly values that were in turn averaged to obtain seasonal and annual estimates for air temperatures. Seasons were defined using the standard meteorological definition: winter = December, January and February; spring = March, April and May; summer = June, July and August, and autumn = September, October and November.

Air temperature trends were also determined using the MK test to assess the probability that a trend exists in the data that is statistically different from zero, and to evaluate its increasing or decreasing slope [41]. Even though the time series has data gaps, the analysis can be performed because the MK test allows for missing data by appropriately reducing the value of the sample size. When a significant trend (P < 0.05) in the air temperature was detected, the magnitude of the slope (*b*) was computed and expressed in °C yr^-1^. Finally, we examined the number of heat waves during winter and early spring (i. e., from December to April), which corresponds to the birth season and the post-weaning fast, respectively. A heat wave was defined as a single day with an average maximum temperature ≥ 25.1°C. This temperature value was used as a threshold because it was determined as the upper critical air temperature for harbour seal (*Phoca vitulina*) dry weaned pups [42].

## Results

### Status of the Baja California population

**Fig 2:**
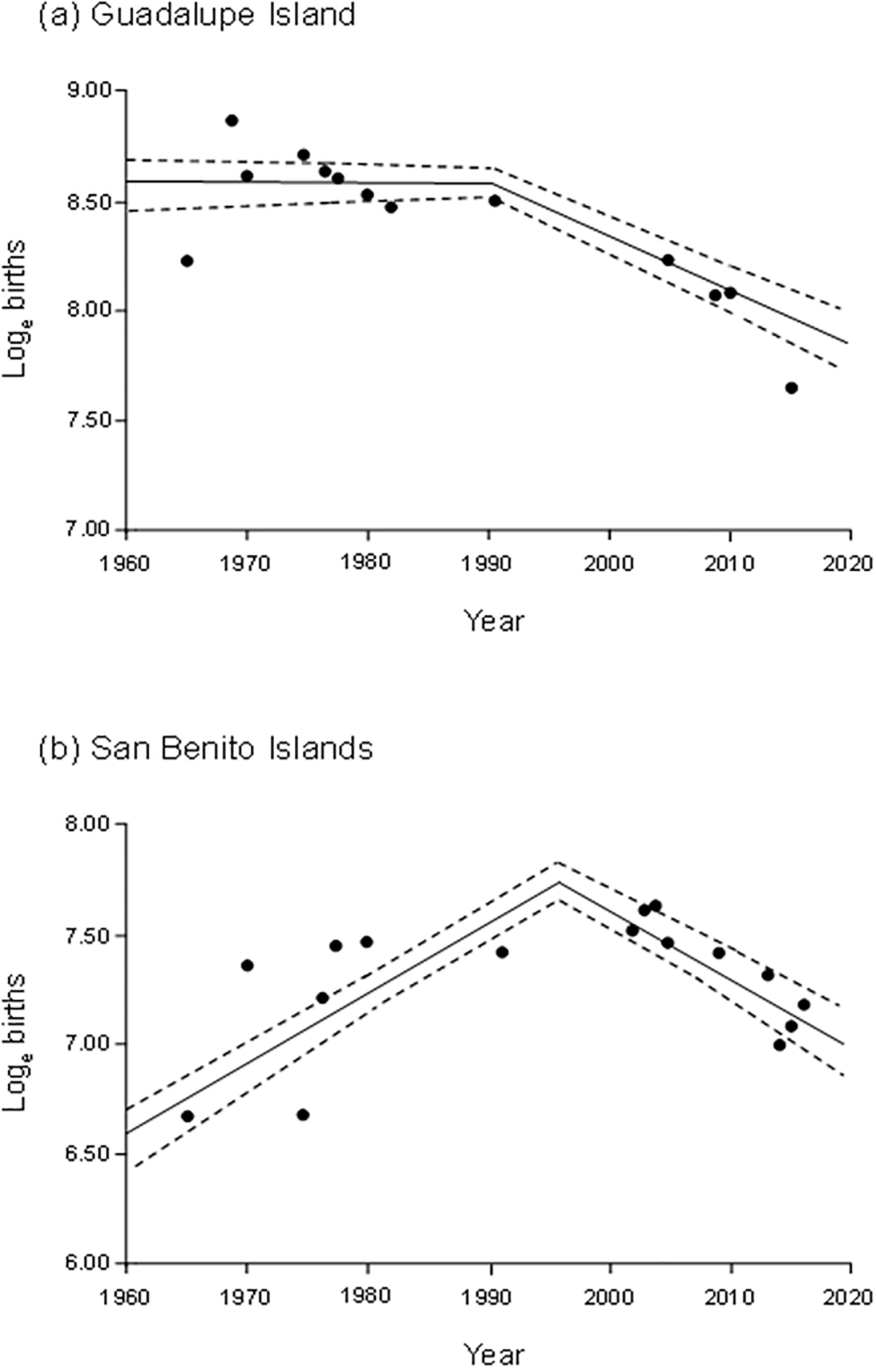
Trends of the northern elephant seal colonies at (a) Guadalupe and (b) San Benito islands. Dotted lines represent the 90% confidence interval.

The optimal breakpoints for the Guadalupe (*n* = 13) and San Benito (*n* = 16) colonies we found to be 1991 and 1997, respectively, and trend analyses showed that both colonies are currently declining (Table 2, Fig 2). The Cedros colony appears to have stabilized since 1991 (Table 2).

**Table 2:** 

Colony	Period	*n*	R^2^	*λ*	CV
Guadalupe Is.	1965–1991	9	<0.01	1.00	0.00
	1992–2015	4	0.86	0.97	-0.45
San Benito Is.	1965–1997	7	0.35	1.03	0.08
	1998–2016	9	0.82	0.97	-0.33
Cedros Is.	1970–2010	8	0.78	1.05	0.12
	1991–2010	4	0.02	1.00	0.01
All colonies	1970–2009	4	0.76	0.99	-0.09

Average annual rate of increase (*λ*) estimates for the Baja California colonies of the northern elephant seal; *n* = number of counts, R^2^ = coefficient of determination, CV = coefficient of variation.

The number of pups in the three major colonies in Baja California in 2009 was estimated to be n = 5,174. Fifty-nine percent of births occurred in the Guadalupe colony, 33% in San Benito, and 8% in Cedros (see Table 1). Forty-one more pups were reported at Coronados Islands (27 pups), Todos Santos Islands (10), San Martín Island (3), and San Jerónimo Island (1) [21] (see Fig 1).

For the period 1970–2009, the average annual rate of increase of the Baja California population was calculated to be 0.99 (Table 2). The multiplicative factor *M* at this *λ* value is 4.31 (95% CI: 3.59–5.02) [18], and hence the population size in 2009 was estimated to be n = 22,300 individuals (95% CI: 18,575–25,974).

### Climate trends

**Fig 3:**
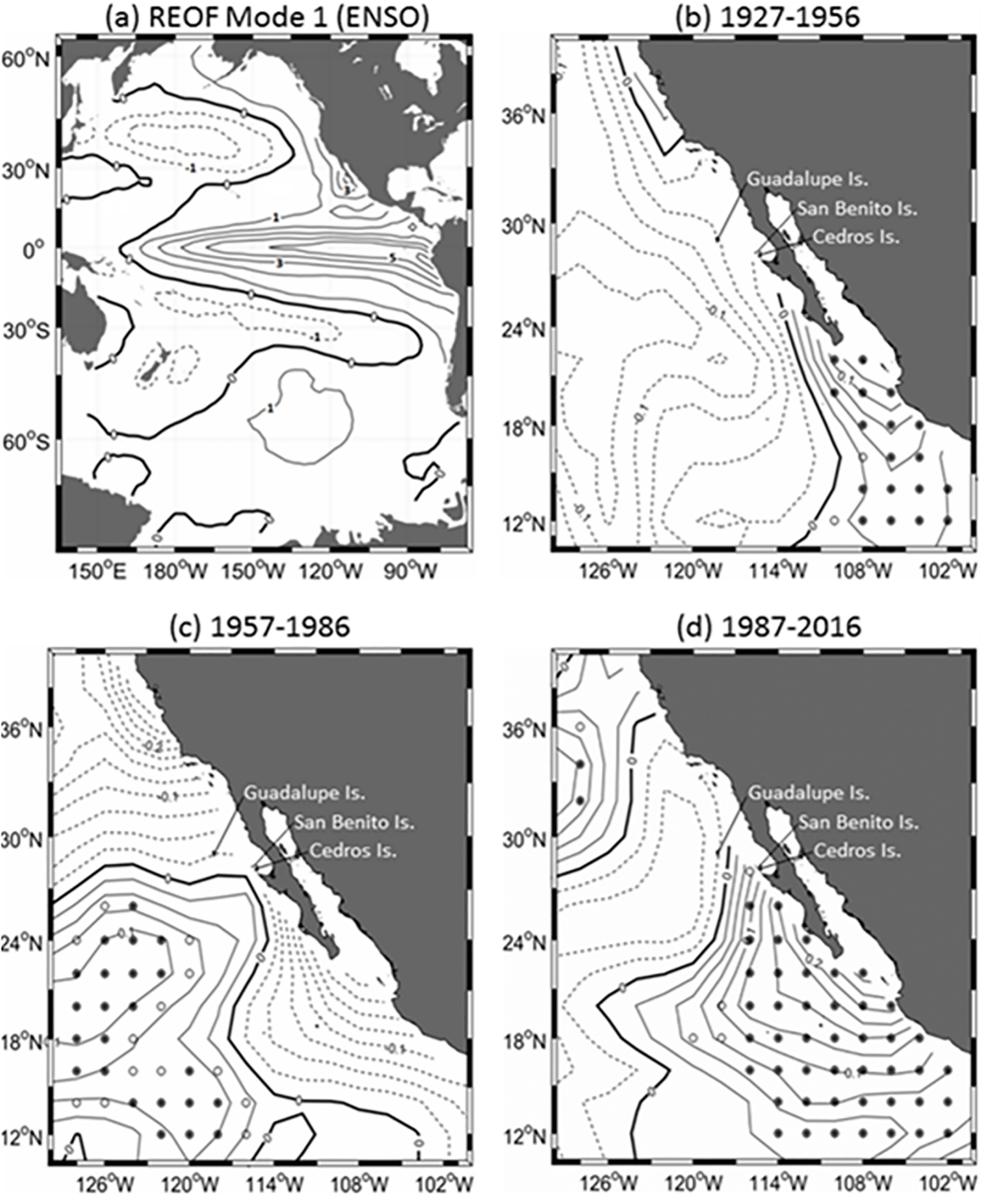
Pacific Ocean SST analysis. (a) Spatial structure of REOF mode 1; units are arbitrary and contour interval is 1 unit. SST trends (°C decade^-1^) estimated over 30 year periods for (b) 1927–1956; (c) 1957–1986; (d) 1987–2016; contour interval is 0.025°C decade^-1^ and open (filled) circles indicate grid points with statistically significant trends at the 95% (99%) confidence level, as determined by the Mann-Kendall non parametric test.

As expected, the dominant mode of variability of the filtered and detrended SST data in the Pacific Ocean was due to the ENSO phenomenon (REOF mode 1, Fig 3a). The expansion coefficients, i.e. the time varying component, of the first mode of our REOF analysis were highly correlated (r = 0.94) with the Multivariate ENSO Index [43], the mode accounted for 55% of the total variance of the SST fields. For any given ENSO event of either sign, the region experienced SST anomalies that are in phase with the equatorial Pacific Ocean and have magnitudes that were roughly half of those that occur at the epicenter of the phenomenon in the ocean near Perú and Ecuador.

The SST analysis (Fig 3) revealed that Pacific Ocean surface waters off Baja California, south of 30°N, have been warming during the last three decades (1987–2016, Fig 3d) at a rate that roughly doubles the rate of increase in global mean SST of 0.06°C decade^-1^ reported to have occurred since 1910 [44]. This recent strong warming trend is in sharp contrast to the cooling that occurred in that region throughout most of the 20th century (Fig 3b and 3c). The first three decades of our study period (1897–1926) also had similar cooling trends.

The signal of the Pacific Decadal Oscillation (PDO) was not isolated in a single mode by our REOF analysis, as was the case with ENSO and the first mode. Rather, decadal variability in our REOF decomposition was spread out between modes 1 and 2; their expansion coefficients correlated with the widely used monthly PDO index [45] with r = 0.63 and r = 0.45, respectively, both at P < 0.05. Correlation of the expansion coefficients of all other modes with the PDO index is significantly smaller. Therefore, decadal variability related to the PDO phenomenon was only partially removed with the subtraction of REOF mode 1 prior to the calculation of the SST trends presented in Fig 3. It is noteworthy however that the PDO was in its negative phase throughout most of the period reflected in Fig 3d [46].

**Fig 4:**
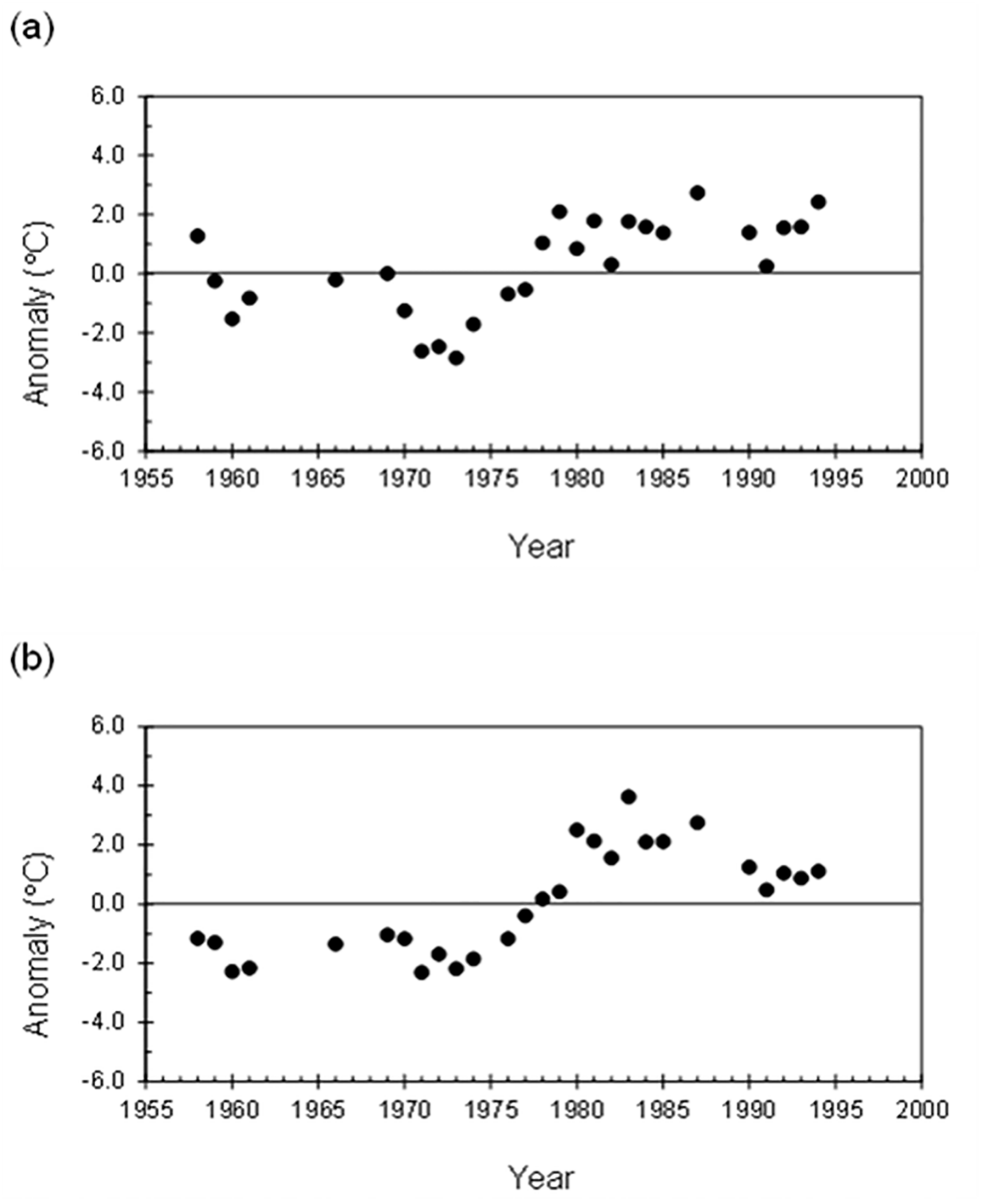
Departures from the 1961–1990 average of the annual (a) mean and (b) minimum air temperature recorded at Cedros Island.

The average annual air temperature for the study period (1957–1995) was 19.8 ± 3.0°C (range: -1.1, 40.2°C). The MK test revealed positive and significant trends in both the annual and seasonal time series of the mean and minimum air temperatures. The maximum temperatures had positive but not significant trends (Table 3). Therefore, over the period, minimum air temperatures increased at a higher rate than the maximum temperatures. The maximum positive trends of the mean temperature were detected during summer (0.10°C yr^-1^) and autumn (0.12°C yr^-1^), but the highest rate of increase of the minimum air temperature was found in the autumn and winter time series (both at 0.15°C yr^-1^). The annual mean temperature increased at a rate of 0.09°C yr^-1^, whereas the minimum temperature increased by 0.12°C yr^-1^ (Table 3). Temperature anomalies relative to the 1960–1991 average revealed that the 1970s were a cold decade, but both the mean and minimum air temperatures have increased since 1978 (Fig 4).

**Table 3:** 

		Mean			Maximum		Minimum		
Season	*n*	*Z*	p	*b*	*Z*	p	*Z*	p	*b*
Winter	32	2.45	0.01	0.06	0.63	0.53	3.32	<0.01	0.15
Spring	30	2.78	0.01	0.08	1.21	0.23	3.75	<0.01	0.13
Summer	27	3.34	<0.01	0.10	1.13	0.26	3.00	<0.01	0.09
Autumn	33	3.98	<0.01	0.12	1.19	0.23	4.35	<0.01	0.15
Annual	27	3.34	<0.01	0.09	1.46	0.14	3.67	<0.01	0.12

Mann-Kendall trend test results (*Z* and p-values), and the magnitude of the slope *b* (°C yr^-1^) for the air temperature series of Cedros Island, 1957–1995; *n* = sample size (years).

One thousand two hundred sixty-one heat waves were recorded during 5,061 days with available data (which represents a frequency of 24.9%) in the winter and early-spring time series of the whole study period. The frequency of the heat waves increased significantly in the period 1978–1995 (32.3%) with respect to 1957–1977 (17.5%) (χ^2^ = 148.7, P < 0.05). The average air temperature also increased, from 16.4 ± 2.7°C (range: –1.1, 32.3°C) in 1957–1977 to 19.0 ± 2.0°C (range: 8.0, 34.0°C) in 1978–1995.

## Discussion

During the early 21st century, the number of northern elephant seals using Baja California islands is decreasing because the two major colonies of Guadalupe and San Benito have experienced population declines, whereas the Cedros colony seems to have stabilized. For 2009, the abundance in Baja California was estimated to be ca. 22,000 individuals. Adding that to the abundance estimated for California in 2010 [18], the total population size of the northern elephant seal at the end of the 2000s would be roughly about 201,000 individuals. Since the numbers in Baja California are declining, and the numbers in California are increasing, the contribution of the Baja California colonies in terms of the number of births decreased from 25% in the early 1990s [23] to 11% in the late 2000s.

The northern elephant seal is protected by the Mexican laws, and all the islands of Baja California are natural areas protected by the Mexican government. Thus, the Baja California population is not subject to any kind of human exploitation, and its natural environment is managed under conservation plans. Moreover, no major mortality events (e.g., due to disease outbreaks) have been detected so far, and incidental mortality in fishing equipment seems to be very low [47]. Thus, environmental factors seem to be the main causes of population fluctuations.

Since the distribution of pinnipeds is influenced by their ability to thermoregulate while on land [42], we propose that the population declines of the Baja California colonies are related to observed climatic change. For land pinnipeds, heat is an environmental threat because of metabolic rate increases and risk of hyperthermia or heat stroke [48]. Northern elephant seals are physiologically well adapted to the cold-waters of the North Pacific, but heat dissipation could be a problem when they are on their temperate and subtropical breeding areas. Like other phocids, they cannot pant and do not have sweat glands, and heat is dissipated through thermal windows [49, 50]. On warm days, elephant seals throw moist sand on their back and move towards the water′s edge [51], but these movements are energetically very costly and can affect the mother-pup relationship during the nursing period [52, 53].

Our SST analysis shows that the surface waters off Baja California have warmed during the last three decades. The recent strong SST warming trend of the habitat off Baja California of the northern elephant seal is in sharp contrast to the weak cooling that occurred in that region throughout most of the 20th century. The region of the Pacific Ocean that is adjacent to the Baja California peninsula is directly affected by ENSO events, and positive El Niño associated ENSO events must therefore constitute a source of significant thermal stress for northern elephant seals. The PDO was in its negative phase throughout most of the period 1987–2016 and its spatial structure is similar to the ENSO mode in that its negative phase implies a cooling effect in the region of interest [46].

The SST and air temperature are in general correlated. In coastal zones of California, changes in minimum air temperatures are driven by SST variability [54, 55]. The annual mean surface air temperature around Baja California islands is in the range 17–20°C [56, 57]. We detected an increment of 0.9°C decade^-1^ for the period 1957–1995, and this warming was mainly caused by the increment in minimum air temperatures. Unfortunately, the lack of recent data makes it impossible to confirm if this trend in air temperature has persisted over the past 20 years. However, it is likely due to the close relationship between SST and air temperature in the study areas. Alternately, heat waves, as defined in this study, are not unusual events in the region, and their frequency during winter and spring has increased since the late 1970s. These events could potentially produce hyperthermia, and are especially dangerous during the birthing season and the post-weaning fast because pups have diminished heat tolerance due to their high mass-specific metabolism [42, 58]. In fact, during an extreme warm winter in the late 2000s, pup mortality at one of the San Benito Islands was >90% in harems without access to the sea [59].

## Conclusions

The results obtained in this study suggest that numbers of northern elephant seals in Baja California colonies are decreasing, and the climate in the region is warming. Since population sizes in the Channel Islands of southern California are increasing [18], we propose that these seals may not be migrating as far south as in previous years, probably in response to the observed temperature increases. Current climate projections predict that the SST and air temperatures will continue to warm during the 21st century, with the strongest warming projected for the tropical and subtropical regions of the Northern Hemisphere [1]. Under these environment scenarios, it is possible that the Baja California colonies will continue to decrease in the future, while the Channel Islands colonies will continue to grow. Further research on the dispersal of northern elephant seals is necessary to confirm this change using, for example, tagged animals. Our results also imply that the colonies of Baja California and California are not demographically isolated, as previously suggested [47], but should be considered and managed as a single population.
